# European Union Should Actively Stimulate and Harmonise Neonatal Screening Initiatives

**DOI:** 10.3390/ijns4040032

**Published:** 2018-11-14

**Authors:** J. Gerard Loeber

**Affiliations:** International Society for Neonatal Screening Office, Burgemeester Fabiuspark 55, 3721CK Bilthoven, The Netherlands; gerard.loeber@gmail.com; Tel.: +31-6-4616-3922

**Keywords:** (recommended) screening panel, policy making, harmonisation, patient advocacy

## Abstract

Neonatal screening programmes have been introduced in almost all European countries. In practice there are large differences, especially in the panel of conditions that are screened for, often without clear reasons. Policy making on a European level is lacking in contrast to the situation in the USA. Professionals have the knowledge to expand the panels but are dependent on policy-makers for the necessary funds. This paper is a call on the EU Commission to take up a role in providing equal access to neonatal screening for all children within the EU.

## 1. Introduction

Neonatal screening, in some countries called newborn screening (NBS), has been recognised as a valuable public health tool in many countries around the world. Based on the work by, e.g., Følling, Penrose and Centerwall in the 20th-century interbellum, Guthrie developed the first relatively easy and cheap assay for the identification of newborn children suffering from phenylketonuria [1]. In the following decade, the development of (radio)immunoassay systems opened the gate for the detection of blood components indicative of congenital hypothyroidism (CH) [2]. These two conditions were just the first of many more. Especially the development of the tandem mass spectrometry in the 1990s led to the possibility of high throughput screening of many newborn children for up to 40–50 different conditions [3,4].

It is self-evident that the implementation of an NBS programme based on modern technologies comes with a certain cost; the apparatus and the necessary manpower are relatively expensive, although this must also be seen in relation to the annual workload of each laboratory. In addition, so-called “multiplexing” methods as tandem mass spectrometry, facilitate the detection of more conditions without a substantial increase in running costs. On the other hand, most of the conditions in the screening panel, if undetected, very often lead to serious health problems, such as mental disabilities with concomitant high health care costs.

NBS is a clear example of a prevention programme with “cost before benefit”. This notion should appeal to politicians and policy-makers in any country or jurisdiction. Everywhere money is tight and choices have to be made. An often-used framework for decision making are the criteria by Wilson and Jungner [5], which, even though they were published 50 years ago, are still valid today.

It would be ideal if policy-makers from different countries who are using these same criteria when judging how to structure their NBS programmes, could come to more or less the same conclusions whether or not to include a certain condition in their screening panel. In practice, this is not the case. Policy-makers give different “weights” to the various factors involved, such as who is the primary beneficiary of the screening system (baby/parent/society), how is the scientific evidence evaluated, and how is the system financed [6,7]. As a consequence, within Europe, the number of conditions screened for ranges from 1 (in Montenegro) to 35 (in Italy) [8,9,10].

Of course, it is understandable that poorer countries have less room for manoeuvring than richer countries. Moreover, the prevalence of a condition may vary, making it a good candidate in one country and an unlikely candidate in another; a well-known and often cited example is phenylketonuria (PKU), which hardly occurs in the Finnish population. Furthermore, the prevalence of certain conditions may increase over time, such as haemoglobinopathies, which have been part of the panels in Mediterranean countries since the 1990s. However, because of recent migration this prevalence is now so high that it was deemed appropriate to be included in the screening panels in the Netherlands, the U. and parts of Belgium.

Unfortunately, there is little interaction and discussion among policy-makers in various countries. Everyone is fixed on their own national situation. There is little willingness to accept the scientific data from other countries that indicate the net benefit of the inclusion of a condition in the screening panel. On the contrary, often the policy-makers require repetition of data collection by the medical professionals within their own country to satisfy the politicians before they decide.

Viewed from a distance, one could imagine that this situation would not apply to the member states of the European Union. After all, the EU, or at least its predecessors European Steel and Coal Community (ECSC) and European Economic (EEC), was established to facilitate interaction and collaboration between and among member states in many fields. The European Treaties have outlined how such collaboration should take place. Yet, health care has been a contentious topic. In the Maastricht and Amsterdam Treaties, Article 129.1 clearly states that *Community action, which shall complement national policies, shall be directed towards improving public health, preventing human illness and diseases, and obviating sources of danger to human health.* However, in the following paragraph, this has been limited immediately to an encouraging role only while leaving the initiative to the member states themselves (principle of subsidiarity): *The Community shall encourage cooperation between the Member States in the areas referred to in this Article and, if necessary, lend support to their action. Member States shall, in liaison with the Commission, coordinate among themselves their policies and programmes in the areas referred to in paragraph 1. The Commission may, in close contact with the Member States, take any useful initiative to promote such coordination. (Art 129.2)* [11,12]. This balancing act has been a major theme also in later EU documents on health care. See, for example, the European Committee of Experts in Rare Diseases (EUCERD) Opinion 2013 [13].

Therefore, for the neonatal screening community, the EU Council recommendations concerning rare diseases in 2009 came as a surprise [14]. Although it is focussed on rare diseases in general, it is very applicable to NBS since virtually all conditions in the NBS panels fall in the category of rare diseases. Subsequently, a call for tender was issued [15] that has led to the financing of an Executive Agency for Health and Concumers (EAHC)-project concerning the practices of newborn screening in European countries, within and outside the EU. The results have been published in a series of publications [16,17,18]. The Expert Opinion Document [18] has been submitted to the EU Commission that referred it to EUCERD. The conclusions of this committee have been documented [13]: Any subsequent action has been left to the member states.

Surprisingly, in other aspects of the fight against rare diseases, European wide collaboration appeared to be possible, such as the development of European Reference Networks (ERNs) in recent years. ERN’s are centres of expertise with knowledge of only one or a limited number of rare diseases focussed on the treatment of patients. The intention is that within the EU, patients can be referred to an ERN even cross-border.

The question is why such collaboration among member states is possible at the back end of the process, i.e., treatment of identified individuals and not at the front end, i.e., the neonatal screening phase.

## 2. Achievements in the USA

As mentioned above neonatal screening started in the USA in the 1960s. To better judge the developing situation in Europe, and in particular in the European Union, it is informative to look at how NBS further developed and evolved in the USA. The USA has a federal government that in many aspects, including health, can give recommendations to the individual states that, in turn, can adopt them or not. Concerning NBS, until the 21st century, the States followed their own policy, based on their own appreciation of scientific evidence as well as learning from practical experience from other states. This led to large variations in the NBS systems between and among states. Some panels contained three conditions, others, more than 40 [19]. In 2002 the American College of Medical Genetics was commissioned to come up with *“recommendations focussed on newborn screening, including but not limited to the development of a uniform condition panel”.* The study, completed in 2005, led to a panel of 29 primary conditions, i.e., those that every state should screen for, and 25 secondary conditions, i.e., those that do not meet screening criteria but are identified anyway because the same markers are abnormal as in the primary conditions. The 29 were called the “recommended uniform screening panel” or RUSP.

The federal Secretary of Health adopted these recommendations. In the following years, the states harmonised their programmes such that every state now works towards full implementation of the RUSP.

New technical possibilities enabled further conditions to be picked up. To judge the value of such new developments a Secretary’s Advisory Committee was created, issuing reports, based on which the RUSP was increased step by step, now comprising 35 conditions [20]. A protocol was developed to ascertain the scientific basis when considering further expansions of the RUSP [20].

## 3. Differences between the USA and Europe

Americans regard themselves as being an inhabitant of the country called the United States of America and can move around in the whole country. A recent estimate is that annually 10–25% of the population moves its household to another state [21]. Moreover, Americans have only a few common languages, English being the most prominent.

Europeans regard themselves as inhabitants of their own country and consider “Europe” to be far away, in the present time even more so than say 20 years ago. Moving around is much more restricted, although in principle there is free movement within the EU. Nevertheless, it is estimated that only 0.4% of European citizens move to another EU country [22]. In Europe, there is a multitude of languages, at least 40 or so.

These factors make Americans aware of what is going on elsewhere in their country, whereas Europeans, in general, have little or no idea of what is happening in neighbouring countries.

It has been mentioned above that in the USA the States receive recommendations from the federal government. In principle, they could ignore theses, but certainly, as regards to neonatal screening, this does not happen often. Parents- and advocacy-groups, such as March of Dimes and Save Babies through Screening, are very active and with a strong voice. They feel that it is unjustified if a newborn infant in State A is screened for a large number of conditions and in another state B for just a handful of conditions. They exert pressure via social networks and newspapers.

In Europe, there are also such advocacy groups, but they often work only within one country. On a European scale, there are umbrella organisations for professionals but not so many for advocacy groups. Thus, this diminishes the possibility of successful lobbying on a larger scale with politicians and policy-makers for harmonisation of neonatal screening panels.

## 4. The Way Forward in Europe and Especially within the EU

It is probably wishful thinking that the policy making concerning neonatal screening systems and the panel of screened conditions within Europe could be structured in a similar way as in the USA, at least within the next couple of years. EU Member States can always invoke the principle of subsidiarity if they are not inclined to collaborate. That should not prevent the EU Commission from strongly issuing recommendations, even if these are not adopted by all member states [23].

It could be argued that the European screening professionals should develop a “European RUSP” before calling upon action from the EU Commission. However, there seems to be no point in repeating what has been done elsewhere. NBS professionals already exchange views on who is doing what and share positive and negative experiences. The International Society for Neonatal Screening (ISNS), with members in almost all European countries as well as in many countries on other continents, facilitates these exchanges by frequently (co-)organising conferences, sending out monthly newsletters and providing information on its website. ISNS was very much involved in the above mentioned EAHC project [15]. ISNS recently teamed up with the International Patient Organisation for Primary Immunodeficiencies (IPOPI) to approach Commission officials as well as Members of the European Parliament to ask for attention for this topic.

It would be of value if patient and advocacy groups in the various countries would also join forces to convince policy-makers and politicians that collaboration saves time and money and that prevention through NBS is cheaper than treating unscreened patients who have developed clinical symptoms.

Finally, all European countries have ratified the UN Convention of the Rights of the Child [24]. Article 24 concerns the right to have optimal health care. Neonatal screening cannot solve all possible health problems, but it can certainly indicate which children in whatever country will need extra attention of the healthcare professionals.

It is high time that the European Commission instruct the Steering Group on health promotion, disease prevention and management of non-communicable diseases [25] to start work on this topic forthwith. The NBS professionals are ready to help!

## References

[1] Guthrie R., Susi A. (1963). A simple phenylalanine method for detecting phenylketonuria in large populations of newborn infants. Pediatrics.

[2] Dussault J.H., Laberge C. (1973). Thyroxine (T4) determination by radioimmunological method in dried blood eluate: New diagnostic method of neonatal hypothyroidism?. Union Med. Can..

[3] Chace D.H., Millington D.S., Terada N., Kahler S.G., Roe C.R., Hofman L.H. (1993). Rapid diagnosis of phenylketonuria by quantitative analysis of phenylalanine and tyrosine in neonatal blood spots using tandem mass spectrometry. Clin. Chem..

[4] Rashed M.S., Rahbeeni Z., Ozand P.T. (1999). Application of electrospray tandem mass spectrometry to neonatal screening. Seminars Perinatol..

[5] Wilson J.M., Jungner Y.G. (1968). Principles and practice of mass screening for disease. Bull. WHO.

[6] Jansen M.E., Metternick-Jones S.C., Lister K.J. (2016). International differences in the evaluation of conditions for newborn blood sot screening; a review of scientific literature and policy documents. Eur. J. Hum. Genet..

[7] Jansen M.E., Lister K.J., van Kranen H.J., Cornel M.C. (2017). Policy making in newborn screening needs a structured and transparent approach. Front. Public Health.

[8] Loeber J.G. (2007). Neonatal screening in Europe; situation in 2004. J. Inher. Metab. Dis..

[9] Therrell B.L., Padilla C.D., Loeber J.G., Khneisser I., Saadallah A., Borrajo G.J.C., Adams J. (2015). Current status of newborn screening worldwide 2015. Seminars Perinatol..

[10] Loeber J.G. Status of neonatal screening in Europe revisited.

[11] Treaty on European Union, Signed at Maastricht on 7 February 1992. Off J Europ Commun 35: C191. http://eur-lex.europa.eu/legal-content/EN/TXT/PDF/?uri=OJ:C:1992:191:FULL&from=EN.

[12] European Union Treaty of Amsterdam, Off J Europ Commun 1997, ISBN 82-828-1652-46986. http://www.europarl.europa.eu/topics/treaty/pdf/amst-en.pdf.

[13] EUCERD Opinion 2013 Newborn Screening in Europe; Opinion of the EUCERD on Potential Areas for European Collaboration. http://www.eucerd.eu/wp-content/uploads/2013/07/EUCERD_NBS_Opinion_Adopted.pdf.

[14] EU Council Recommendation of 8 June 2009 on an action in the field of rare diseases (2009/C 151/02). http://ec.europa.eu/chafea/documents/health/prague-rd-council-recommendation_en.pdf.

[15] EU-Executive Agency for Health and Consumers Call for Tender “Evaluation of Population Newborn Screening Practices for Rare Disorders in Member States of the European Union” EHC/2009/Health/09. http://old.iss.it/binary/cnmr4/cont/TechnicalSpecifications.pdf.

[16] Loeber J.G., Burgard P., Cornel M.C., Rigter T., Weinreich S.S., Rupp K., Hoffmann G.F., Vittozzi L. (2012). Newborn screening programmes in Europe; arguments and efforts regarding harmonization. Part 1–From blood spot to screening result. J. Inher. Metab. Dis..

[17] Burgard P., Rupp K., Lindner M., Haege G., Rigter T., Weinreich S.S., Loeber J.G., Taruscio D., Vittozzi L., Cornel M.C. (2012). Newborn screening programmes in Europe; arguments and efforts regarding harmonization. Part 2–From screening laboratory results to treatment, follow-up and quality assurance. J. Inher. Metab. Dis..

[18] Cornel M.C., Rigter T., Weinreich S.S., Burgard P., Hoffmann G.F., Lindner M., Loeber J.G., Rupp K., Taruscio D., Vittozzi L. (2014). Expert Opinion document Newborn screening in Europe. Based on the EU Tender “Evaluation of population newborn screening practices for rare disorders in Member States of the European Union”. Eur. J. Hum. Genet..

[19] Watson M.S., Mann M.Y., Lloyd-Puryear M.A., Rinaldo P., Howell R.R. (2006). Newborn screening: Toward a uniform screening panel and system—Executive summary. Pediatrics.

[20] Advisory Committee on Heritable Disorders in Newborns and Children. https://www.hrsa.gov/advisory-committees/heritable-disorders/rusp/index.html.

[21] Avric. http://avrickdirect.com/homedata/?p=31.

[22] Eurostat. https://ec.europa.eu/eurostat/statistics-explained/index.php/Migration_and_migrant_population_statistics/nl.

[23] Addressing the high burden and significant unmet needs in phenylketonuria (PKU). Proceedings of the European Parliament Policy Roundtable.

[24] UN Convention of the Rights of the Child. https://www.ohchr.org/en/professionalinterest/pages/crc.aspx.

[25] EU Steering Group on Health Promotion Disease Prevention and Management of Non-Communicable Diseases. https://ec.europa.eu/health/non_communicable_diseases/steeringgroup_promotionprevention_en.

